# Data on self-awareness, self-determination, and self-efficacy of opioid-dependent patients receiving methadone treatment before and after getting individual psycho-educational (i-SEAZ) intervention

**DOI:** 10.1016/j.dib.2020.105586

**Published:** 2020-04-18

**Authors:** E.M. Engku Kamarudin, W.S. Wan Sulaiman, N.H. Sarnon, A.S. Amin

**Affiliations:** National University of Malaysia, Malaysia

**Keywords:** Self-awareness, Self-determination, Self-efficacy, Psycho-education, Methadone treatment

## Abstract

The readiness to change among drug addicts is a key strength for successful treatment. Self-awareness, self-determination, and self-efficacy have been identified as the fundamentals of readiness that should be embraced by drug addicts while in treatment. In this article, the shared data were applied to assess the effect of individual psycho-educational intervention based on integrated self-awareness and self-determination theories (i-SEAZ) on self-efficacy amongst opioid-dependent patients undergoing methadone treatment (MT). The effectiveness of the i-SEAZ module was evaluated in a total of 75 opioid-dependent MT participants from five Methadone Clinics under the Ministry of Health, Malaysia located across Klang Valley. The experimental group consisted of 38 participants who received 10 sessions of individual i-SEAZ alongside MT, whereas 37 participants of the control group only received MT. The shared data were collected through three questionnaires, namely Scale for Self-Consciousness Assessment (SSCA), Treatment Motivation Questionnaire (TMQ), and General Self Efficacy (GSE). Data collection was performed twice; the first instance was two weeks prior to initiation of i-SEAZ (pretest), and the second was two weeks post completion of i-SEAZ (posttest). The extracted data were precisely represented in terms of means and standard deviations (SDs).

Specifications table

**Table:** 

Subject	Psychology
Specific subject area	Substance Abuse Psychological Treatment
Type of data	Tables and Figure
Acquisition of data	The data were collected using a pretest-posttest design from 75 participants divided into two groups. The questionnaire included a demographic profile, along with The Scale for Self-Consciousness Assessment (SSCA), Treatment Motivation Questionnaire (TMQ), and General Self Efficacy (GSE).
Data format	Raw Data
Parameters for data collection	A total of 75 participants were selected randomly from five Methadone Clinics under the purview of Ministry of Health (MOH), Malaysia. The sample size was determined based on the views of Hair et al. (1), who proposed a sample of five subjects for each variable of analysis as the minimal limit, while the most acceptable method of determination was a ratio of 10:1 (10 subjects for a variable). Schreiber et al. (2006) claimed that each parameter should have at least 10 participants. The G*Power program was used to perform sample calculations since it takes into account the effect of size and statistical advantage. The participants were divided into experimental and control groups, using the matched-pair method.
Description of data collection	Data collection was performed twice: August 2018; two weeks prior to the initiation of i-SEAZ (pretest), and December 2018; two weeks after the completion of i-SEAZ intervention (posttest)
Data source location	Five Methadone Clinics located in Klang Valley, Malaysia were involved. The government facilities met the inclusion criteria of the sample, as the area had the most prevalent Methadone patients in Malaysia. The facilities have been identified as among the earliest MT projects to be undertaken by the MOH. To be eligible for methadone treatment, the patients must meet the DSM-5 criteria for opioid use disorder and diagnosed by a Medical Officer.
Data accessibility	The raw data files were provided by the Data in Brief Dataverse. All other data are reported in this article.

## 1 Value of the data

Addiction is defined as a brain disease (5). This has led to further development in the role of psychology in assisting drug addicts in the recovery process. These data are useful in describing the effect of the i-SEAZ module on self-awareness, self-determination, and self-efficacy among opioid-dependent patients in Methadone Clinics.
The data addresses the gap in available information regarding profiling of opioid-dependent patients and the need for psychological treatment of addiction.
Opioid-dependent patients can benefit from these data to enhance their self-awareness, self-determination, and self-efficacy towards recovery.
These data can be used for further experimental endeavours in the domain of evidence-based psychological treatment.
The data in this article suggest that self-awareness, self-determination, and self-efficacy of opioid-dependent patients can be improved via individual psycho-educational intervention.

## 1 Data description

The subjects were divided into two groups: control (*n* = 37) and experimental (*n* = 38). Both groups had more male than female participants. In both groups, more than 30% of the participants were between 41 and 50 years old. In the control group, the age group of 61–70 years had the fewest subjects (5.4%), while in the experimental group, the age group of 21–30 years had the fewest subjects (5.3%). Half of the subjects in both groups were either married or widowed. The data revealed that the smoke method was more popular in the control group, while the intravenous method using injections was more popular in the experimental group. Most of the participants in both groups had used illicit drugs besides heroin. The first use of heroin among participants was recorded as early as 12 years old and as late as 41 years old, while the average age range at the time of first use was 18–23 years. The data revealed that before they had begun the Methadone treatment, subjects from both groups had attempted and failed to quit drug abuse without treatment. Table 1 presents the demographic profile of the participants.

**Table 1:** Demographic profile of participants.

Measures		Group			
		Control (*N* = 37)		Experimental (*N* = 38)	
*Demographic*		*N*	*(%)*	*N*	*(%)*
Gender	Male	36	97.3	36	94.7
	Female	1	2.7	2	5.3
Age	21–30 years	4	10.8	2	5.3
	31–40 years	12	32.4	11	28.9
	41–50 years	14	37.8	14	36.8
	51–60 years	5	13.5	7	18.4
	61–70 years	2	5.4	4	10.5
Status	Single	15	40.5	16	42.1
	Married	17	45.9	15	39.5
	Widow/Widower	5	13.5	7	18.4
Occupation	Unemployed	8	21.6	3	7.9
	Odd jobs	14	37.8	15	39.5
	Employed	1	2.7	3	7.9
	Business	14	37.8	17	44.7
Duration of addiction	0–10 years	12	32.4	13	34.2
	11–20 years	15	40.5	13	34.2
	21–30 years	10	27	12	31.5
Use of other illicit drugs	Yes	32	86.5	35	92.1
	No	5	13.5	3	7.9
Heroin administration	Smoke	23	62.2	15	39.5
	Intravenous	10	27	22	57.9
	Snort	4	10.8	1	2.6
Start of heroin abuse (Age)	12–17 years	10	27	7	18.4
	18–23 years	19	51.4	17	44.7
	24–29 years	6	16.2	11	29
	30–35 years	2	5.4	2	5.3
	36–41 years	0	0	1	2.6
Previous attempt to quit	Yes	24	64.9	17	44.7
	No	13	35.1	21	55.3

Table 2 presents the mean and SD values of opioid-dependent patients’ self-awareness before and after i-SEAZ intervention. The mean pretest scores of self-awareness for the control and the experimental group were 3.34 and 3.37, respectively; while the mean posttest scores were 3.49 and 3.85, respectively.

**Table 2:** Self-awareness scores before and after the intervention (*N* = 75).

Group	n	Pretest		Posttest	
		Mean	SD	Mean	SD
Control	37	3.34	.52	3.49	.49
Experiment	38	3.37	.41	3.85	.52

Table 3 presents the mean and SD values of opioid-dependent patients’ self-determination before and after the i-SEAZ intervention. The mean pretest scores of self-determination for the control and the experimental group were 4.56 and 4.57, respectively; while the mean posttest scores were 5.38 and 5.62, respectively.

**Table 3:** Self-determination scores before and after the intervention (*N* = 75).

Group	n	Pretest		Posttest	
		Mean	SD	Mean	SD
Control	37	4.56	.85	5.38	.43
Experiment	38	4.57	.83	5.62	.49

Table 4 tabulates the mean and SD values of opioid-dependent patients’ self-efficacy before and after the i-SEAZ intervention. The mean pretest scores of self-efficacy for the control and the experimental group were 2.67 and 2.69, respectively; while the mean posttest scores were 2.23 and 3.14, respectively.

**Table 4:** Self-efficacy scores before and after the intervention (*N* = 75).

Group	n	Pretest		Posttest	
		Mean	SD	Mean	SD
Control	37	2.67	.35	2.23	.21
Experiment	38	2.69	.32	3.14	.20

## 2 Experimental design, materials and methods

A pretest-posttest design with two groups was employed for this data collection by involving patients receiving Methadone treatment at five MOH-affiliated Methadone Clinics in Klang Valley. A total of 75 participants were selected by the random sampling method and divided into two groups (experimental and control) using the matched pair method. The inclusion criteria for the sample are listed below:

1. The patient is above 18 years old.
2. The patient has undergone at least three months of Methadone treatment.
3. The researchers have obtained consent and cooperation for voluntary intervention.
4. The patient has no chronic mental illness, such as schizophrenia and bipolar disorder.
5. If the patient has a chronic infectious disease, such as HIV / AIDS and Tuberculosis (TB), they must be admitted until stable, under the care and supervision of a Medical Officer.

These criteria were devised by the researchers to minimize the incidence of dropout and to adhere to sample presence during the intervention.

In the beginning, the study participants were divided into experimental and control groups with 50 participants per group, using the matched-pair method based on the four steps proposed by Ismail (7). This method ascertained that both groups were equal and homogeneous in terms of achievement scores prior to treatment. This procedure was performed on a single-blind basis, whereby the participants were unsure of the group to which they belonged. Participants from the experimental group attended the weekly individual intervention sessions conducted by a trained facilitator for 10 weeks. The time allotted per session was 60 min; hence, the total duration of intervention over 10 weeks was 600 min. Participants from the control group were omitted from the intervention, but received intervention after the completion of the experiment. A pretest was conducted two weeks before the i-SEAZ module intervention began, whereas the posttest was executed two weeks after the intervention. Upon completion of this period, the valid number of participants analysed was 75 (control group, *n* = 37; experimental group, *n* = 38). The reduction had been due to several unavoidable factors, such as transfer of patients to other rehabilitation centres, patients defaulting, patients being arrested by the authorities, patients going missing, and outlier cases.

Initially, the demographic profile of the participants was gathered, including gender, age, marital status, employment, duration of addiction, drug administration, age at the time of first consumption of illicit drugs, and previous effort to quit addiction without treatment. Three academicians established content validity of the demographic questionnaire.

The second questionnaire was the Scale for Self-Consciousness Assessment (SSCA), a self-assessment tool that measures self-awareness. This questionnaire was developed by Mylonas, Veligekas, Gari, and Kontaxopoulou (2) and consists of 24 items scored on a five-point Likert scale. The scale has two dimensions, namely public and private self-consciousness. Each dimension has four factors, namely appearance, social fit, self-reflectiveness, and self-knowledge. Content validity of the instrument was evaluated by a panel of three experts and the percentage of consensus exceeded 80%. The reliability of the questionnaire was determined in a pilot test on 90 participants; the Cronbach's alpha was found to be 0.94.

The third questionnaire was the *Treatment Motivation Questionnaire (*TMQ) developed by Ryan, Plant, and O'Malley (4) . The TMQ is composed of 26 questions to be rated on a seven-point Likert scale (1 = not at all; 7 = very true), arranged in four subscales (External Reasons, Internal Reasons, Confidence of Treatment, and Help Seeking). The questionnaire was translated into the Malay language and was back-translated into the English language, as suggested by the WHO translation process. In a pilot test performed on 90 subjects, the Cronbach's alpha reliability of the questionnaire was found to be 0.82.

The fourth instrument was the General Self-Efficacy (GSE) questionnaire. The GSE contains 10 items to assess positive self-esteem in coping with various severe events in one's life. The original scale developed in 1981 by Matthias Jerusalem and Ralf Schwarzer consisted of 20 items and was later modified into a shorter version in 1995. The questionnaire has been translated into the Malay language and validated in a number of studies within the context of Malaysia. The items are rated on a four-point Likert scale, with the options being (1) Strongly Disagree, (2) Disagree, (3) Agree, and (4) Strongly Agree. The GSE questionnaire displayed exceptional reliability with the Cronbach's alpha value exceeding 0.80, as reported in prior local studies (see Baba et al., 8; 3; 6).

After gaining permission from the research chancellery of MOH and attaining consent from the participants, the questionnaires were distributed to the participants at an appropriate venue after they were briefed about the purpose of the data collection. The subjects were evaluated two weeks before the i-SEAZ intervention and two weeks after. After that, the data gathered were analysed.

All data were treated with full confidentiality. They were analysed and summarised using descriptive statistics, including absolute (n) and relative (%) frequencies for categorical variables, and mean and SD for self-awareness, self-determination, and self-efficacy before and after the i-SEAZ intervention among the selected opioid-dependent patients.

## Appendix Supplementary materials

Image, application 1

Image, application 2

Image, application 3
