# Supplementary material for: Data on self-awareness, self-determination, and self-efficacy of opioid-dependent patients receiving methadone treatment before and after getting individual psycho-educational (i-SEAZ) intervention
Source: Data Brief. 2020 Apr 18;30:105586. doi: 10.1016/j.dib.2020.105586 (PMC7182707; doi:10.1016/j.dib.2020.105586)
Supplement: Supplementary file 1 [file mmc1.pdf]

General Self-Efficacy Scale (GSE)

About: This scale is a self-report measure of self-efficacy.

Items: 10

Reliability:
Internal reliability for GSE = Cronbach's alphas between .76 and .90

Validity:
The General Self-Efficacy Scale is correlated to emotion, optimism, work satisfaction. Negative coefficients were found for depression, stress, health complaints, burnout, and anxiety.

Scoring:

	Not at all true	Hardly true	Moderately true	Exactly true
All questions	1	2	3	4

The total score is calculated by finding the sum of the all items. For the GSE, the total score ranges between 10 and 40, with a higher score indicating more self-efficacy.

References:
Schwarzer, R., & Jerusalem, M. (1995). Generalized Self-Efficacy scale. In J. Weinman, S. Wright, & M. Johnston, *Measures in health psychology: A user's portfolio. Causal and control beliefs* (pp. 35-37). Windsor, UK: NFER-NELSON.

General Self-Efficacy Scale (GSE)

	Not at all true	Hardly true	Moderately true	Exactly true
1. I can always manage to solve difficult problems if I try hard enough	☐	☐	☐	☐
2. If someone opposes me, I can find the means and ways to get what I want.	☐	☐	☐	☐
3. It is easy for me to stick to my aims and accomplish my goals.	☐	☐	☐	☐
4. I am confident that I could deal efficiently with unexpected events.	☐	☐	☐	☐
5. Thanks to my resourcefulness, I know how to handle unforeseen situations.	☐	☐	☐	☐
6. I can solve most problems if I invest the necessary effort.	☐	☐	☐	☐
7. I can remain calm when facing difficulties because I can rely on my coping abilities.	☐	☐	☐	☐
8. When I am confronted with a problem, I can usually find several solutions.	☐	☐	☐	☐
9. If I am in trouble, I can usually think of a solution	☐	☐	☐	☐
10. I can usually handle whatever comes my way.	☐	☐	☐	☐
