# Supplementary material for: Data on self-awareness, self-determination, and self-efficacy of opioid-dependent patients receiving methadone treatment before and after getting individual psycho-educational (i-SEAZ) intervention
Source: Data Brief. 2020 Apr 18;30:105586. doi: 10.1016/j.dib.2020.105586 (PMC7182707; doi:10.1016/j.dib.2020.105586)
Supplement: Supplementary file 2 [file mmc2.pdf]

Scale for Self-Consciousness Assessment

Mylonas, Veligekas, Gari, & Kontaxopoulou (2012)

To the researchers: Please mask the name of the scale ("Personality assessment" or something equivalent should appear instead) during data collection; also mask the authors' names.

Please rate each of the following personality statements using the scale provided; it will take you no
longer than 5 minutes. Please rate all items and report the first answer that comes to mind.
Confidentiality of your answers is ensured and scores are solely used for statistical analysis purposes
and as a part of broader anonymous databases pursuing scientific and theoretical goals.

Rating scale (*mark what holds true for you*):

5 = always true for me
4 = many times true for me
3 = sometimes true and sometimes not true
2 = few times true for me
1 = never true

SSCA1 I evaluate myself after every action of mine 5 4 3 2 1
SSCA3 In my collaborations, I am interested in maintaining friendly relationships 5 4 3 2 1
SSCA5 Prior to my actions I check with my desires 5 4 3 2 1
SSCA18 I am aware of my desires and I can describe them 5 4 3 2 1
SSCA12 Prior to my actions I check with my pursuits 5 4 3 2 1
SSCA14 I am interested in what impression I make to other people 5 4 3 2 1
SSCA15 Prior to my actions I check with my needs 5 4 3 2 1
SSCA16 When with friends, I am interested in being honest 5 4 3 2 1
SSCA21 I am interested in how other people evaluate me 5 4 3 2 1
SSCA26 When with friends, I am interested in being understanding 5 4 3 2 1
SSCA29 I am concerned about how other people evaluate me 5 4 3 2 1
SSCA30 When with friends, I am interested in being helpful 5 4 3 2 1
SSCA23 I am concerned about what other people think about me 5 4 3 2 1
SSCA32 I act carefully and with precision 5 4 3 2 1
SSCA35 I am interested in how I present myself to other people 5 4 3 2 1
SSCA36 I can easily describe my thoughts 5 4 3 2 1
SSCA37 I am concerned about the impression I make to other people 5 4 3 2 1
SSCA39 I can describe my emotions 5 4 3 2 1
SSCA38 I am interested in my physical appearance at my work-place 5 4 3 2 1
SSCA10 Prior to my actions I check with my motives 5 4 3 2 1
SSCA42 I am interested in what other people think about me 5 4 3 2 1
SSCA7 Prior to my actions I check with my specific traits 5 4 3 2 1
SSCA41 I think about myself and I feel I have a deep knowledge of me 5 4 3 2 1
SSCA9 I criticize myself on the basis of previous experience 5 4 3 2 1

Sex: ❑ Male ❑ Female Age (in years): _________

Thank you very much for collaboration.

Green items: PR1 factor
Blue items: PU1 factor
Black items: PR2 factor
Red items: PU2 factor
