# Supplementary material for: Data on self-awareness, self-determination, and self-efficacy of opioid-dependent patients receiving methadone treatment before and after getting individual psycho-educational (i-SEAZ) intervention
Source: Data Brief. 2020 Apr 18;30:105586. doi: 10.1016/j.dib.2020.105586 (PMC7182707; doi:10.1016/j.dib.2020.105586)
Supplement: Supplementary file 3 [file mmc3.pdf]

Treatment Motivation Questionnaire

Ryan, Plant, and O'Malley (1995)

Treatment Motivation Questionnaire (TMQ), and the Treatment Self-Regulation Questionnaire (TSRQ). The TMQ has two motivation factors: (a) internal--which includes both introjected and identified items; and (b) external--which is merely external items.

The TMQ Scale

This questionnaire concerns people's reasons for entering treatment and their feelings about treatment. Participation is voluntary, so you do not have to fill it out if you don't want to.Different people have different reasons for entering treatment, and we want to know how true each of these reasons is for you. Please indicate how true each reason is for you, using the following scale:

1	2	3	4	5	6	7
not at all true			somewhat true			very true

A. I came for treatment at the clinic because:
1. I really want to make some changes in my life.
2 I won't feel good about myself if I don't get some help.
3. I was referred by the legal system.
4. I feel so guilty about my problem that I have to do something about it.
5. It is important to me personally to solve my problems.

B. If I remain in treatment it will probably be because:
6. I'll get in trouble if I don't.
7. I'll feel very bad about myself if I don't.
8. I'll feel like a failure if I don't.
9. I feel like it's the best way to help myself.
10. I don't really feel like I have a choice about staying in treatment.
11. I feel it is in my best interests to complete treatment.

C. Rate each of the following in terms of how true each statement is for you.
12. I came to treatment now because I was under pressure to come.
13. I am not sure this program will work for me.
14. I am confident this program will work for me.
15. I decided to come to treatment because I was interested in getting help.
16. I'm not convinced that this program will help me stop drinking.
17. I want to openly relate with others in the program.
18. I want to share some of my concerns and feelings with others.

19. It will be important for me to work closely with others in solving my problem.
20. I am responsible for this choice of treatment.
21. I doubt that this program will solve my problems.
22. I look forward to relating to others who have similar problems.
23. I chose this treatment because I think it is an opportunity for change.
24. I am not very confident that I will get results from treatment this time.
25. It will be a relief for me to share my concerns with other program participants.
26. I accept the fact that I need some help and support from others to beat my problem.

Scoring the TMQ. Calculate the four subscale scores by averaging the responses for item in that subscale. The external reasons and internalized reasons are the subscales that relate most directly to self-determination theory.

External Reasons: 3, 6, 10, 12
Internalized Reasons: 1, 2, 4, 5, 7, 8, 9, 11, 15, 20, 23
Help Seeking: 17, 18, 19, 22, 25, 26
Confidence: 13(R), 14, 16(R), 21(R), 24(R) **Note**: An (R) after items in the Confidence subscale means that the item should be reverse scored before averaging it with other items in the subscale. To do that, subtract the person's response from 8. Thus,for example, a 3 becomes a 5. This way, a higher score means more confidence in treatment.

References concerning the TMQ

Ryan, R. M., Plant, R. W., & O'Malley, S. (1995). Initial motivations for alcohol treatment: Relations with patient characteristics, treatment involvement and dropout. *Addictive*
Behaviors, 20, 279-297.
Zeldman, A., Ryan, R. M., & Fiscella, K. (1999). Attitudes, beliefs and motives in addiction recovery. Unpublished manuscript, University of Rochester.
